# Identifying Pleiotropic Genes in Genome-Wide Association Studies for Multivariate Phenotypes with Mixed Measurement Scales

**DOI:** 10.1371/journal.pone.0169893

**Published:** 2017-01-12

**Authors:** James J. Yang, L. Keoki Williams, Anne Buu

**Affiliations:** 1 School of Nursing, University of Michigan, Ann Arbor, Michigan, United States of America; 2 Department of Internal Medicine, Henry Ford Health System, Detroit, Michigan, United States of America; 3 The Center for Health Policy and Health Services Research, Henry Ford Health System, Detroit, Michigan, United States of America; 4 Department of Health Behavior and Biological Sciences, University of Michigan, Ann Arbor, Michigan, United States of America; University of the Chinese Academy of Sciences, CHINA

## Abstract

We propose a multivariate genome-wide association test for mixed continuous, binary, and ordinal phenotypes. A latent response model is used to estimate the correlation between phenotypes with different measurement scales so that the empirical distribution of the Fisher’s combination statistic under the null hypothesis is estimated efficiently. The simulation study shows that our proposed correlation estimation methods have high levels of accuracy. More importantly, our approach conservatively estimates the variance of the test statistic so that the type I error rate is controlled. The simulation also shows that the proposed test maintains the power at the level very close to that of the ideal analysis based on known latent phenotypes while controlling the type I error. In contrast, conventional approaches–dichotomizing all observed phenotypes or treating them as continuous variables–could either reduce the power or employ a linear regression model unfit for the data. Furthermore, the statistical analysis on the database of the Study of Addiction: Genetics and Environment (SAGE) demonstrates that conducting a multivariate test on multiple phenotypes can increase the power of identifying markers that may not be, otherwise, chosen using marginal tests. The proposed method also offers a new approach to analyzing the Fagerström Test for Nicotine Dependence as multivariate phenotypes in genome-wide association studies.

## Introduction

Since the first genome-wide association study (GWAS) [1], more than 2,000 loci have been identified to be significantly associated with one or more complex traits [2]. In the early days, researchers have focused on genes associated with well defined functions or specific traits. A systematic review showed that many loci are actually associated with multiple traits [3]. This motivates researchers to study pleiotropy which is a condition in which a single gene affects multiple traits. A well-known example published on Nature is a GWAS involving 107 phenotypes that identified multiple pleiotropy genes [4]. From a statistical point of view, for complex diseases such as substance use disorders, a gene usually affects multiple traits and yet the effect size on each trait is very small. A GWAS using marginal association tests tends to have low power to detect these small effects. However, if a test can model the association between this gene and multivariate phenotypes *simutaneously*, the statistical power would be greatly increased [5].

When all the multivariate phenotypes are continuous, our team [6] recently conducted a comprehensive review of relevant statistical methods commonly used in the field including the principal component analysis (PCA), the multivariate analysis of variance (MANOVA), the generalizing estimating equations (GEE), the trait-based association test involving the extended Simes procedure (TATES), and the classical Fisher combination test. In the same study, we proposed a new method that relaxes the unrealistic independence assumption of the classical Fisher combination test and is computationally efficient. Our simulations also showed that the proposed method has higher power than existing methods while controlling the type I error rate.

Most of the existing methods that we previously reviewed and compared were designed for continuous multivariate phenotypes. However, it is pretty common in practice that multivariate phenotypes are measured in different scales (i.e. non-commensurate). For example, in substance abuse research, the early onset use of a substance and the lifetime exposure to a substance are both important traits and yet, the former is usually measured as a binary outcome whereas the latter tends to be a continuous or ordinal outcome [7]. The methodological challenge of modeling the association between a gene and non-commensurate phenotypes is that there does not exist a multivariate distribution for mixed data types.

There are two approaches that can handle bivariate phenotypes with one continuous variable and one binary variable [8]. The first approach is to model the bivariate phenotypes by factoring the joint distribution into the product of conditional and marginal distributions [9]. The complexity of this approach, however, increases exponentially when the number of phenotypes increases. The other approach is based on a latent class model with the assumption that conditioning on the latent variable, the bivariate phenotypes are independent. Hence, one can write the joint distribution as a product of the two conditional distributions. Nevertheless, one critical issue with this approach is that the parameters in the latent class model are not identifiable without some constraints as demonstrated in the example in Teixeira-Pinto and Normand (2009) [8].

In comparison to the methods reviewed above, meta-analysis is a more flexible alternative for handling different types of phenotype data [10]. The first step of this approach is to carry out separate analyses for different types of data (e.g. generalized linear models for continuous, binary, or ordinal phenotypes). The *p*-values from these analyses are later aggregated into a summary statistic. If this summary statistic is more extreme than the critical value, the significance of association between a SNP and multivariate phenotypes is declared. However, the key restriction of this approach is that the sample used to derived the *p*-value for one phenotype cannot be used to derive the *p*-value for another phenotype. If we partition the entire sample to multiple subsets for the purpose of meta-analysis, the statistical power would be greatly reduced.

In this study, we extend the Fisher combination function method originally designed for continuous multivariate phenotypes [6] to handle mixed continuous, binary, or ordinal multivariate phenotypes. This new method is also applicable to any number of phenotypes while controlling the type I error rate. Furthermore, the majority of computation time for the proposed method is used to calculate the marginal *p*-values, whereas the rest of computation time for the Fisher combination function is minimal regardless of the number of phenotypes involved. Therefore, this method is highly effective for exploring multiple combinations of multivariate phenotypes with minimal extra computation time.

This paper is organized as follows. In the next section, we review our previous work on continuous multivariate phenotypes and propose an extension of the previous method to handle multivariate phenotypes with mixed measurement scales. We also show that our proposed method controls the type I error rate. Because the proposed method requires estimation of the correlations for various combinations of phenotypes, we propose the estimation methods in the Estimation of the Correlation between Mixed Phenotypes section. In the Simulation Studies section, we present the results of simulation studies that evaluate the proposed method in terms of the accuracy of correlation and variance estimation, the type I error rate and statistical power. The Real Data Analysis section presents the results of statistical analysis on the Study of Addiction: Genetics and Environment (SAGE) data to demonstrate the applications in the substance abuse field. Discussions and concluding remarks are presented in the Discussions section.

## Methods

### Previous Work on Continuous Multivariate Phenotypes

In this section, we review our previous work on continuous multivariate phenotypes [6] so that the readers have sufficient background information to understand the proposed method in the next section. For each individual *i* (= 1, …, *N*), let *Z*_*ig*_ (= 0, 1, 2) be the number of reference alleles for SNP *g* (= 1, …, *G*), and *R*_*ij*_, (*j* = 1, …, *M*) be the *j*th phenotypes. To simplify notation, we define ***Z***^(*g*)^ = (*Z*_1*g*_, …, *Z*_*Ng*_) as the *g*th genotypes; and ***R***^(*j*)^ = (*R*_1*j*_, …, *R*_*Nj*_) as the *j*th phenotype. Let *p*_*gj*_ be the *p*-value from the marginal test of the association between ***Z***^(*g*)^ and ***R***^(*j*)^. Thus, for the *g*th genotype, we have a collection of *p*-values {*p*_*g*1_, …, *p*_*gM*_} for the *M* phenotypes. The purpose of this article is to construct an efficient and powerful method for testing the association between the *g*th SNP and the multivariate phenotypes {***R***^(1)^, …, ***R***^(*M*)^} using these marginal *p*-values {*p*_*g*1_, …, *p*_*gM*_}.

Given {*p*_*g*1_, …, *p*_*gM*_}, the Fisher combination statistic is defined as

$$S^{\left(g\right)}=\sum_{j=1}^{M}-2\log\left(p_{gj}\right).$$

There are other choices of combination functions but the Fisher combination is chosen because of its asymptotic optimality [11, 12]. Based on the Fisher combination statistic, we may conduct a permutation test to examine the association between the *g*th SNP and the multivariate phenotypes {***R***^(1)^, …, ***R***^(*M*)^}. Although the permutation test is unbiased and asymptotically equivalent to the best parametric tests [13], it is extremely time consuming and thus not feasible for carrying out a whole genome association test that would require performing more than 10^6^ permutations.

When the phenotypes {***R***^(1)^, …, ***R***^(*M*)^} follow a multivariate normal distribution, the test statistic *S*^(*g*)^ is a sum of chi-squared statistics under the null hypothesis of no association between the genotype and phenotypes. Since multivariate phenotypes are correlated (i.e. the *p*-values in *S*^(*g*)^ are correlated), the null distribution of *S*^(*g*)^ follows a gamma distribution with the shape parameter *κ* and the scale parameter *ν* [14, 15]. That is,

$$\begin{array}{c} E\left[S^{\left(g\right)}\right]=\kappa \nu ,\\ Var\left[S^{\left(g\right)}\right]=\kappa \nu^{2}. \end{array}$$

If we can estimate *κ* and *ν*, we can calculate the *p*-value of *S*^(*g*)^ using the gamma distribution rather than the permutation method. This will greatly improve the computation efficiency.

Because −2 log(*p*_*gj*_) follows a chi-squared distribution with 2 degrees of freedom, we have

$$\kappa \nu =E\left[S^{\left(g\right)}\right]=2M,$$ (1)

$$\kappa \nu^{2}=Var\left[S^{\left(g\right)}\right]=4M+\sum_{j\neq j^{\prime }}cov\left(-2\log\left(p_{gj}\right),-2\log\left(p_{gj^{\prime }}\right)\right).$$ (2)

Here, the covariance between the *p*-value of the *j*th phenotype and the *j*′th phenotype, *cov*(−2 log *p*_*gj*_, −2 log *p*_*gj*′_), is a function of the correlation between ***R***^(*j*)^ and ***R***^(*j*′)^ [6, 15]. Define *ρ*_*jj*′_ to be the correlation between ***R***^(*j*)^ and ***R***^(*j*′)^. Our previous work showed that *cov*(−2 log (*p*_*gj*_), −2 log (*p*_*gj*′_)) can be accurately estimated as

$$cov\left(-2\log\left(p_{gj}\right),-2\log\left(p_{gj^{\prime }}\right)\right)\approx \sum_{l=1}^{5}c_{l}\rho_{jj^{\prime }}^{2l}-\frac{c_{1}}{N}{\left(1-\rho_{jj^{\prime }}^{2}\right)}^{2},$$ (3)

where *c*_1_ = 3.9081, *c*_2_ = 0.0313, *c*_3_ = 0.1022, *c*_4_ = −0.1378 and *c*_5_ = 0.0941. Note that this approximation is very accurate as the maximum difference is less than 0.001. Thus, we can efficiently estimate *κ* and *ν* using Eqs (1) and (2) with the *cov*(⋅) in Eq (2) substituted by the right-hand side of Eq (3).

### The Proposed Method for Multivariate Phenotypes with Mixed Measurement Scales

The method reviewed in the previous section is based on the strong assumption that the multivariate phenotypes follow a multivariate normal distribution. Although we have demonstrated its robustness against a long-tail multivariate distribution in a simulation study, it may not be applicable to multivariate phenotypes with mixed measurement scales [6]. In this section, we extend the method to handle mixed continuous, binary, or ordinal phenotypes.

The proposed method is a two-phase approach: the first phase conducts a marginal test for each phenotype; and the second phase uses the Fisher combination function to combine the p-values from the first phase and conducts a multivariate test. The prerequisite for the multivariate test to be valid is that the marginal tests generating *p*_*gj*_ are unbiased [16, 17]. In order to meet this criterion, we propose to conduct the marginal tests based on the measurement scales of the phenotypes: using the linear regression for continuous phenotype; the logistic regression for binary phenotypes; and the cumulative logit model for ordinal phenotypes [18]. We propose to use those regression models in Phase 1 not only because they are unbiased but also because we can add covariates in the models to increase the accuracy of testing as well as principal components to correct for population stratification [19].

Once we obtain *p*-values {*p*_1*g*_, …, *p*_*Mg*_} in Phase 1, the second phase is to calculate the correlation *ρ*_*jj*′_ between the phenotypes ***R***^(*j*)^ and ***R***^(*j*′)^, so that we can estimate *cov*(−2 log(*p*_*gj*_), −2 log (*p*_*gj*′_)) using Eq (3). There are two issues that we need to address. First, we need to find appropriate methods for estimating *ρ*_*jj*′_ when one or both phenotypes are binary or ordinal. This issue is dealt with in detail in the next section where various estimation methods of correlation are presented for all possible combinations of measurement scales. The second issue is to find the relationship between *cov*(−2 log(*p*_*gj*_), −2 log(*p*_*gj*′_)) and $\sum_{l=1}^{5}c_{l}\rho_{jj^{\prime }}^{2l}-\frac{c_{1}}{N}{\left(1-\rho_{jj^{\prime }}^{2}\right)}^{2}$ when the correlation *ρ*_*jj*′_ estimated from non-continuous data is used in Eq (3). We propose a latent response model to address this issue.

Assume that the response ***R***^(*j*)^ is viewed as a partial or full observation of a continuous *latent response* R*^(*j*)^. When the phenotype is continuous, R*^(*j*)^ is fully observed and equal to ***R***^(*j*)^. However, when the phenotype is binary or ordinal, a certain value of ***R***^(*j*)^ is observed when R*^(*j*)^ falls within an unknown fixed threshold. The binary phenotype is treated as a special case when there is only one threshold. We further assume that (R*^(1)^, …, R*^(*M*)^) follows a multivariate normal distribution and the correlation between R*^(*j*)^ and R*^(*j*′)^ is *ρ*_*jj*′_. Let $p_{gj}^{†}$ and $p_{gj\prime }^{†}$ be the *p*-values derived from observed binary or ordinal phenotypes. We propose to plug ${\hat{\rho }}_{jj^{\prime }}$ in Eq (3) to estimate the true covariance $cov(-2\log\left(p_{gj}^{†}\right),-2\log\left(p_{gj^{\prime }}^{†}\right))$. However, under the latent response model, this approach is actually estimating $cov(-2\log\left(p_{gj}^{*}\right),-2\log\left(p_{gj^{\prime }}^{*}\right))$, where $p_{gj}^{*}$ and $p_{gj^{\prime }}^{*}$ are the *p*-values calculated from the latent variables R*^(*j*)^ and R*^(*j*′)^. Since the binary or ordinal ***R***^(*j*)^ and ***R***^(*j*′)^ are derived from the continuous R*^(*j*)^ and R*^(*j*′)^, the covariance between ***R***^(*j*)^ and ***R***^(*j*′)^ is smaller:

$$\begin{array}{c} cov\left(-2\log\left(p_{gj}^{†}\right),-2\log^{†}\left(p_{gj^{\prime }}\right)\right)\leq cov\left(-2\log\left(p_{gj}^{*}\right),-2\log\left(p_{gj^{\prime }}^{*}\right)\right). \end{array}$$

Thus, this approach will over-estimate the covariance of the observed test statistic. In other words, we conservatively estimate the variance of *S*^(*g*)^ so that the type I error is controlled. In the Simulation Studies section, we conduct a simulation study to examine the difference between the true covariance and our estimates.

## Estimation of the Correlation between Mixed Phenotypes

In this section, we specify various estimation methods of correlation for all possible combinations of measurement scales. Table 1 summarizes the classification of correlation coefficients based on the variable types. We define the following simplified notations for ease of interpretation. Suppose (*U*_*i*_, *V*_*i*_)′, *i* = 1, …, *n*, are independent and identical bivariate normal random variables and the correlation between *U*_*i*_ and *V*_*i*_ is *ρ*. We would like to estimate *ρ* but either *U*_*i*_, *V*_*i*_ or both are latent variables. The observed data may be binary (coded 0 or 1) or ordinal (coded as positive integers) depending on the practical situation. In the following sections, we describe different approaches to estimate *ρ* depending on the types of observed variables. Subscripts or superscripts may be omitted for convenience.

**Table 1:** Different types of correlation coefficients when the variables *X* and *Y* are continuous, binary, or ordinal.

		*X*		
		Continuous	Binary	Ordinal
*Y*	Continuous	Kendall	Biserial	Polyserial
	Binary		Tetrachoric	Polychoric
	Ordinal			Polychoric

### Kendall Correlation: Continuous-Continuous

Suppose we observe $X_{i}^{c}$ and $Y_{i}^{c}$ where $X_{i}^{c}=U_{i}$ and $Y_{i}^{c}=V_{i}$. To estimate the correlation coefficient *ρ*, the natural estimator is Pearson’s sample correlation *r*_*p*_. Although Pearson’s sample correlation is an asymptotically unbiased estimator of *ρ* and the variance of *r*_*p*_ reaches the Cramer-Rao lower bound as the sample size increases, it tends to over or underestimate *ρ* when the sample distribution of ${\left(X_{i}^{c},Y_{i}^{c}\right)}^{\prime }$ deviates from the bivariate normal distribution or the regression of $Y_{i}^{c}$ on $X_{i}^{c}$ (or vice versa) is nonlinear [20]. Moreover, it cannot handle incomplete data. Our previous work demonstrated that Kendall *τ* is robust against these problems and thus is chosen to estimate the correlation between continuous variables [6]. Kendall *τ* is defined as

$$\begin{array}{c} \tau =\frac{K_{c}-K_{d}}{n(n-1)/2}, \end{array}$$

where *K*_*c*_ is the number of concordant pairs ($X_{i}^{c}$ and $Y_{j}^{c}$), and *K*_*d*_ is the number of discordant pairs. To use kendall’s *τ* to estimate *ρ*, we can use this transformation [21, 22]:

$$\begin{array}{c} r_{k}=\text{sin}\left(\frac{\pi \tau }{2}\right). \end{array}$$

Thus, we adopt *r*_*k*_ when both phenotypes are continuous.

### Biserial Correlation: Continuous-Binary

Suppose we observed $Y_{i}^{c}=V_{i}$ and $X_{i}^{b}=I_{\left[U_{i}\geq C\right]}$, where *I* is an indicator function and *C* is a fixed unknown threshold. Pearson proposed the sample *biserial* correlation to estimate *ρ* [23]. However, its absolute value was shown to exceed 1 when |*ρ*| > 0.798 [24]. Brogden considered the situation when *ρ* > 0 and proposed a better biserial estimator [25]:

$$\begin{array}{c} r_{Brogden}=\frac{\sum_{i}Y_{i}^{c}X_{i}^{b}-n{\bar{X}}^{b}{\bar{Y}}^{c}}{\sum_{i=1}^{\sum X_{i}}Y_{\left(n-i+1\right)}^{c}-n{\bar{X}}^{b}{\bar{Y}}^{c}}, \end{array}$$

where $Y_{\left(1\right)}^{c}\leq Y_{\left(2\right)}^{c}\leq \ldots \leq Y_{\left(n\right)}^{c}$. Note that *r*_*Brogden*_ ≤ 1 but *r*_*Brogden*_ may be less than −1 when *ρ* < 0. Lord further modified Borgden’s estimator as Lord’s estimator [26]:

$$\begin{array}{c} r_{L}=\left\{\begin{array}{cc} r_{Brogden} & \text{if}\;r_{Brogden}\geq 0\\ r_{Brogden}^{†} & \text{if}\;r_{Brogden}<0, \end{array}\right. \end{array}$$

where $r_{Brogden}^{†}=-r_{Brogden}\left(X_{i}^{b},-Y_{i}^{c}\right)$. The Lord’s biserial estimator ensures that *r*_*L*_ is always between −1 and 1 and were shown by simulations to be more efficient in comparison to other estimators [27]. Thus, in this study we adopt *r*_*L*_ to estimate *ρ* for continuous and binary variables.

### Tetrachoric Correlation: Binary-Binary

Suppose we observe $X_{i}^{b}=I_{\left[U_{i}>C_{1}\right]}$ and $Y_{i}^{b}=I_{\left[V_{i}>C_{2}\right]}$ for unknown thresholds *C*_1_ and *C*_2_. Let the proportions in 2 × 2 contingency tables be *p*_11_, *p*_12_, *p*_21_, *p*_22_. Define the marginal proportions as *p*_*x*_ = *p*_11_ + *p*_12_ and *p*_*y*_ = *p*_11_ + *p*_21_. Since the underlying variables follow a bivariate normal distribution, Pearson proposed the following likelihood function to find the *tetrachoric* correlation for *ρ* [28]:

$$\begin{array}{c} L\left(h,k,\rho \right)=\frac{1}{2\pi \sqrt{1-\rho^{2}}}\int_{k}^{\infty }\int_{h}^{\infty }exp\left(-\frac{x^{2}-2\rho xy+y^{2}}{2(1-\rho^{2})}\right)dxdy \end{array}$$

where *h* = Φ^−1^(*p*_*x*_), *k* = Φ^−1^(*p*_*y*_), and Φ^−1^ is the inverse of the standard normal distribution function. The maximum likelihood estimate (MLE) for *ρ* is derived by solving

$$\begin{array}{c} L\left(h,k,\rho \right)=p_{11}. \end{array}$$

In this study, we adopt the computational algorithm developed by Good (2006) [29] to calculate this MLE.

### Polyserial Correlation: Continuous-Ordinal

Let $Y_{i}^{c}=V_{i}$ and $X_{i}^{o}=t\;\text{if}\;\zeta_{t-1}\leq U_{i}\;<\;\zeta_{t}$, for a positive integer *t* (≥ 2), where −∞ = *ζ*_0_ < *ζ*_1_ < … < *ζ*_*t*−1_ < *ζ*_*t*_ = ∞. Cox derived the likelihood function of observations ${\left(X_{i}^{o},Y_{i}^{c}\right)}^{\prime }$ using the following factorization [30]:

$$\Pi_{i=1}^{n}f\left(X_{i}^{o}=x_{i},Y_{i}^{c}=y_{i}\right)=\Pi_{i=1}^{n}f\left(y_{i}\right)Pr\left[x_{i}|y_{i}\right]$$ (4)

where $Y_{i}^{c}$ is a normal random variable and the conditional probability of $X_{i}^{o}|Y_{i}^{c}$ is

$$\begin{array}{c} Pr\left[x_{i}|y_{i}\right]=\Phi \left(\theta_{j}\right)-\Phi \left(\theta_{j-1}\right), \end{array}$$

where Φ is the standard normal distribution function and $\theta_{j}=\frac{\zeta_{j}-\rho \left(y_{i}-\mu \right)/\sigma }{{\left(1-\rho^{2}\right)}^{1/2}}.$ The MLE is obtained by maximizing the likelihood function in Eq (4). In this study, we adopt the computational algorithm developed by Olsson et al. (1982) [31] to calculate the MLE.

### Polychoric Correlation: Binary-Ordinal or Ordinal-Ordinal

Suppose that the variables we observe are both ordinal. That is, the relation between *X*_*i*_ and *U*_*i*_ is

$$\begin{array}{llll} X_{i} & = & 1 & \text{if }-\infty =\zeta_{0}\leq U_{i}<\zeta_{1}\\ X_{i} & = & 2 & \text{if }\zeta_{1}\leq U_{i}<\zeta_{2}\\ & & \vdots & \\ X_{i} & = & R & \text{if }\zeta_{R-1}\leq U_{i}<\zeta_{R}=\infty . \end{array}$$

The relation between *Y*_*i*_ and *V*_*i*_ is similar to this. Pearson and Pearson (1922) [32] proposed the polychoric correlation which can be applied to handle ordinal-ordinal and binary-ordinal (special case) variables. If we arrange the data as a two-way contingency table with observed frequencies *n*_*ij*_, *i* = 1, …, *R* and *j* = 1, …, *C*. Define *π*_*ij*_ as the probability of observing *n*_*ij*_. Then the likelihood function is proportion to

$$\begin{array}{c} L\left(\rho \right)\propto \Pi_{i=1}^{R}\Pi_{j=1}^{C}\pi_{ij}^{n_{ij}}, \end{array}$$

where *π*_*ij*_ = Φ(*ζ*_*i*_, *ξ*_*j*_) − Φ(*ζ*_*i*−1_, *ξ*_*j*_) − Φ(*ζ*_*i*_, *ξ*_*j*−1_) + Φ(*ζ*_*i*−1_, *ξ*_*j*−1_). Note that Φ(⋅, ⋅) is the standard bivariate normal distribution function which is also a function of *ρ*. Olsson developed an algorithm to derive MLE which was shown by a simulation study to have a small bias with the variance being close to the theoretical value [33]. We adopt this algorithm to calculate the polychoric correlation.

## Results

Three simulation studies were conducted to evaluate (1) the accuracy of the proposed estimation methods for correlations between phenotypes; (2) the accuracy of the proposed estimation method for the variance of test statistic; and (3) the type I error rate and statistical power of the proposed multivariate test in comparison to competing methods. A real data analysis was used to identify pleiotropic genes for the risk of nicotine dependence.

### Accuracy of the Estimation of Correlation between Phenotypes

We conducted a simulation study to evaluate the accuracy of different correlation estimation methods for mixed continuous, binary, and ordinal data described in the previous section. Because most genome-wide association studies contain more than 1,000 subjects, we simulated 1,000 individuals. For each individual, we simulated a pair of continuous phenotypes from bivariate normal random variables. The correlation *ρ* for the bivariate normal distribution ranges from −0.9 to 0.9. The binary variables were derived from the continuous variables by dividing the observed values into two parts. Similarly, the ordinal variables were derived from the continuous variables by dividing their values into five parts. We later created six different combinations among continuous, binary or ordinal variables. For each simulated pair of phenotypes, we estimated its correlation using the corresponding method described in Estimation of the Correlation between Mixed Phenotypes section. We repeated the process 10,000 times to calculate the mean and standard deviation for each configuration. The simulation results in Table 2 show that all the proposed methods estimate the true *ρ* well. The confidence intervals cover the true *ρ* in all situations. The standard deviations are small. Even the largest standard deviation, which occurred with both phenotypes being binary and the correlation being around zero, is about 0.05. Therefore, the accuracy level is high for all of the proposed correlation estimation methods.

**Table 2:** Simulation results for the correlation estimation based on Kendall’s *τ*, biserial, polyserial, tetrachoric, or polychoric correlation. The choice of correlation methods depends on the measurement scale. The values of *ρ* ranges from −0.9 to 0.9. The correlation estimates and standard deviations for various methods are calculated based on 10,000 replications.

*ρ*	Continuous-Continuous	Continuous-Binary	Continuous-Ordinal	Binary-Binary	Binary-Ordinal	Ordinal-Ordinal
−0.9	−0.8999 (0.0066)	−0.9003 (0.0112)	−0.9009 (0.0082)	−0.8999 (0.0159)	−0.9007 (0.0149)	−0.9004 (0.0120)
−0.8	−0.7997 (0.0124)	−0.8002 (0.0185)	−0.8006 (0.0136)	−0.8000 (0.0249)	−0.8007 (0.0213)	−0.8008 (0.0160)
−0.7	−0.6999 (0.0172)	−0.7004 (0.0238)	−0.7007 (0.0183)	−0.7000 (0.0315)	−0.7009 (0.0269)	−0.7008 (0.0206)
−0.6	−0.6000 (0.0218)	−0.6004 (0.0287)	−0.6004 (0.0225)	−0.6002 (0.0374)	−0.6009 (0.0312)	−0.6008 (0.0252)
−0.5	−0.4999 (0.0253)	−0.5001 (0.0324)	−0.5005 (0.0263)	−0.5000 (0.0418)	−0.5009 (0.0350)	−0.5006 (0.0287)
−0.4	−0.4000 (0.0281)	−0.4005 (0.0351)	−0.4005 (0.0289)	−0.3998 (0.0453)	−0.4005 (0.0380)	−0.4005 (0.0311)
−0.3	−0.3001 (0.0302)	−0.3004 (0.0371)	−0.3004 (0.0310)	−0.3003 (0.0477)	−0.3005 (0.0407)	−0.3005 (0.0333)
−0.2	−0.2004 (0.0317)	−0.2010 (0.0388)	−0.2005 (0.0327)	−0.2010 (0.0493)	−0.2004 (0.0418)	−0.2009 (0.0352)
−0.1	−0.1006 (0.0330)	−0.1008 (0.0398)	−0.1008 (0.0337)	−0.1008 (0.0509)	−0.1010 (0.0428)	−0.1011 (0.0359)
0.0	−0.0000 (0.0331)	0.0002 (0.0397)	0.0001 (0.0339)	−0.0001 (0.0506)	−0.0004 (0.0428)	0.0002 (0.0364)
0.1	0.0998 (0.0324)	0.0999 (0.0392)	0.0998 (0.0331)	0.1000 (0.0499)	0.0999 (0.0420)	0.1000 (0.0354)
0.2	0.1996 (0.0317)	0.2003 (0.0390)	0.1998 (0.0323)	0.1997 (0.0494)	0.1995 (0.0414)	0.2000 (0.0348)
0.3	0.2999 (0.0298)	0.3002 (0.0370)	0.3003 (0.0304)	0.3001 (0.0477)	0.3004 (0.0398)	0.3006 (0.0326)
0.4	0.3993 (0.0279)	0.3996 (0.0352)	0.4000 (0.0287)	0.3994 (0.0449)	0.4003 (0.0372)	0.4003 (0.0311)
0.5	0.4997 (0.0249)	0.5004 (0.0319)	0.5001 (0.0259)	0.5001 (0.0416)	0.5005 (0.0347)	0.5006 (0.0284)
0.6	0.6000 (0.0217)	0.6006 (0.0285)	0.6004 (0.0226)	0.6000 (0.0377)	0.6003 (0.0314)	0.6009 (0.0249)
0.7	0.6999 (0.0172)	0.7004 (0.0237)	0.7006 (0.0184)	0.6999 (0.0317)	0.7005 (0.0269)	0.7008 (0.0206)
0.8	0.8000 (0.0124)	0.8004 (0.0184)	0.8008 (0.0137)	0.8003 (0.0250)	0.8006 (0.0215)	0.8011 (0.0162)
0.9	0.8997 (0.0066)	0.9001 (0.0113)	0.9007 (0.0082)	0.8996 (0.0160)	0.9005 (0.0152)	0.8997 (0.0112)

### Accuracy of the Estimation of Variance for Test Statistic

**Fig 1:**
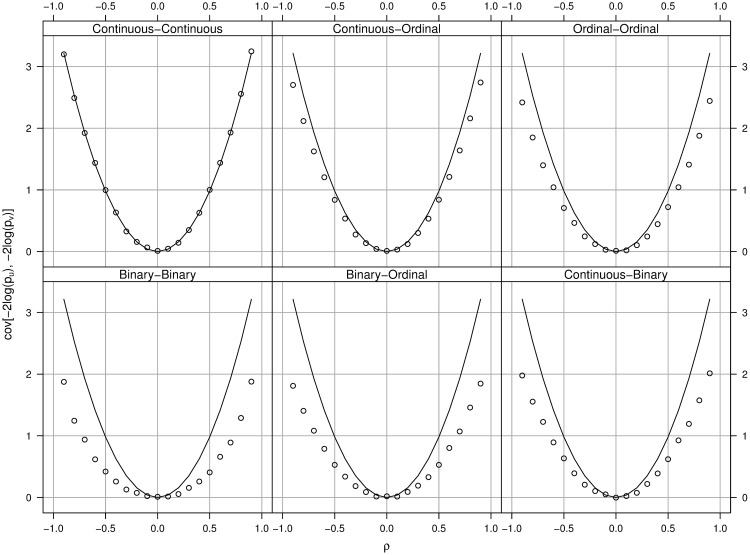
The relationship between the covariance *cov*[−2*log*(*p*_*u*_), −2*log*(*p*_*v*_)] and the correlation *ρ*. The title in each panel indicates the types of data simulated. The solid curve in each panel corresponds to our covariance estimates using Eq (3). The dotted curves are the true covariances calculated from the simulated data.

We conducted a simulation study to evaluate the relative accuracy of the proposed variance estimation method when the phenotypes are a mixture of continuous, binary or ordinal variables. The simulation was based on 1,000 simulated individuals. Given the correlation *ρ* ranging from −0.9 to 0.9, we simulated bivariate normal random variables (*U*, *V*)′ for each individual. The binary and ordinal variables were generated from *U* or *V* following the same procedure as the simulation study described in the previous section. We also independently simulated the genotypes *Z* of all individuals with the minor allele frequency 0.5. We used the linear regression to test the association between a genotype and a continuous phenotype; the logistic regression for a binary phenotype; and the cumulative logit model for an ordinal phenotype. The process was repeated 10,000 times so that we have 10,000 pairs of *p*-values for each of the six types of combination (i.e. continuous-continuous, continuous-ordinal, ordinal-ordinal, binary-binary, binary-ordinal, and continuous-binary). Based on these simulated *p*-values, we can calculate their covariance which is considered the *true* covariance for the purpose of comparison. Since the values of *ρ* are known in the experiment, they were plugged in Eq (3) to produce the *estimate* of the covariance based on the proposed method. Fig 1 summarizes the simulation results with the solid curves being the estimates and the dotted curves being the true covariances. The findings from this simulation study are summarized as follows:

1. The covariances depend on the values of *ρ*. When *ρ* = 0, the covariance is zero. When |*ρ*| increases, the covariance increases.
2. The true covariance is always less than or equal to the covariance estimate using the right-hand side of Eq (3).
3. When both phenotypes are continuous (the top-left panel), the covariance estimates match the true covariances.
4. When one or both phenotypes are not continuous, the covariance estimates tend to over-estimate the true covariances.
5. The difference between the true covariances and the estimates varies across different data types. For example, the difference for continuous-ordinal data is relatively small. On the other hand, the difference for binary-binary data is the largest among all six combinations.

In summary, the results indicate that our proposed estimation method tends to slightly over-estimate the true covariance. For example, when *ρ* = 0.5 and both the observed phenotypes are binary, our estimate of *Var*[*S*^(*g*)^] is 9.9956 which over-estimates the target value of 8.813 by 13%. Nevertheless, when one or both phenotypes are continuous or ordinal, the difference is much smaller. Furthermore, because our approach conservatively estimates the variance of the test statistic *S*^(*g*)^, the resulting type I error rate is controlled.

### Type I Error Rate and Statistical Power of the Proposed Multivariate Test

A simulation study was conducted to evaluate the performance of the proposed method in terms of the type I error rate and statistical power. We considered a pleiotropic gene model in which multivariate phenotypes were modeled as a function of the candidate gene with varied effect sizes. For each individual, we simulated the genotype *Z* = (0, 1, 2) based on the minor allele frequency (MAF) which is uniformly distributed on [0.1, 0.5]. Therefore, *Z* represents the number of reference alleles for a SNP. A total of 100 individuals were generated. The latent phenotypes were simulated from multivariate normal (MVN) random variables. We considered (*V*_1_, *V*_2_, …, *V*_6_)′ ∼ *MVN*((*μ*_1_, *μ*_2_, …, *μ*_6_)′, Σ) where the diagonal elements of Σ are 1 and the off diagonal elements of Σ are *ρ*. The value of *μ*_*i*_(*i* = 1, …, 6) was defined as

$$\mu_{i}=\left\{\begin{array}{cc} -e_{i} & \text{if}\;Z=0\\ 0 & \text{if}\;Z=1\\ e_{i} & \text{if}\;Z=2, \end{array}\right.$$

where *e*_*i*_ is the genetic effect size.

The observed phenotypes (*U*_1_, *U*_2_, …, *U*_6_)′ with mixed measurement scales were derived from the simulated latent variables (*V*_1_, *V*_2_, …, *V*_6_)′. Let *U*_*i*_ = *V*_*i*_(*i* = 1, 2) represent the continuous measurements. By dividing *V*_*i*_(*i* = 3, 4) into two intervals with the cut-off value *C*, we derived binary measurements: *U*_*i*_ = 1 if *V*_*i*_ > *C* or *U*_*i*_ = 0 if *V*_*i*_ ≤ *C*(*i* = 3, 4). For the ordinal scale, we divided *V*_*i*_(*i* = 5, 6) into five intervals using 4 cut-off points and assigned the values of 1 to 5 to *U*_*i*_(*i* = 5, 6) accordingly.

The values of *ρ* were set at 0, 0.35 and 0.75 to represent independent, moderate dependent, or highly dependent multivariate phenotypes. We also manipulated the values of *e*_1_, …, *e*_6_ to be 0, 0.5, 0.7, or 0.9 to represent different genetic effect sizes. Note that the configuration of all *e*_1_, …, *e*_6_ being equal to zero represents the null condition of no genetic effect.

We compared the proposed method (labelled as *Mixed*), with three alternative approaches. When there was no method available for handling mixed phenotypes, people tended to analyze them as phenotypes in the same measurement scale. One commonly adopted approach is to dichotomize each of (*U*_1_, *U*_2_, …, *U*_6_)′ and carry out the analysis with the marginal *p*-values derived from a logistic regression model (labelled as *Dichotomous*). Another naive approach is to treat each of (*U*_1_, *U*_2_, …, *U*_6_)′ as continuous measurements and carry out the analysis with the marginal *p*-values derived from a linear regression model (labelled as *Continuous*). We also compared our method with the ideal situation when the analysis is conducted on the latent phenotypes (*V*_1_, *V*_2_, …, *V*_6_)′. This approach is labelled as *Latent* and serves as our gold standard.

The empirical type I error rates were set at 10^−4^. In order to evaluate the performance of competing methods in terms of controlling the type I error, we carried out 10^6^ replications under the null conditions. For the conditions with non-zero effect sizes, 10^4^ replications are sufficient to show the differences in power. The simulation results are shown in Table 3. The findings are summarized as follows:

1. Based on 10^6^ iterations, a half of the width of 95% confidence interval is 0.00139. Therefore, under the null conditions when all the effect sizes are zero (*e*_*i*_ = 0, *i* = 1, …, 6), all the four methods control the type I error.
2. When some or all effect sizes (*e*_*i*_) are nonzero, the power of all methods decreases as the correlation *ρ* increases. The decrease in power is expected because highly correlated phenotypes contain less information than phenotypes with low correlations.
3. Among all four methods, the *Latent* has the highest power and the *Dichotomous* has the lowest power. When mixed measurement scales are observed, dichotomizing all observed measurements could reduce the power by half.
4. The power of the proposed method (*Mixed*) is very close to that of the *Latent* which is the gold standard. Because the true values of latent phenotypes are unknown in real situations, this result demonstrates that our proposed method can provide an efficient and powerful way to conduct multivariate testing with phenotypes in mixed measurements in practice.
5. If we treat all mixed measurement scales as continuous variables and apply the proposed method to it (i.e. the *Continuous*), the power is close to that of the proposed method. In some situations, it had even higher power than the proposed method. However, modeling binary or ordinal responses as continuous variables is both mathematically and practically questionable. For instance, in the binary case, the mean response value is within 0 and 1 but the predicted values from a linear regression model would cover the entire real line [18]. Furthermore, Guisan and Harrell (2000) [34] provided four reasons why applying a linear regression model is statistically incorrect when the outcome is ordinal. Therefore, we do not recommend the use of the *Continuous* approach.
6. In comparison to the situation when all phenotypes are associated with the pleiotropic gene, the power of all methods tends to be reduced when a half of the phenotypes are not associated with the gene (*e*_1_ = *e*_3_ = *e*_5_ = 0). Even when the nonzero effect sizes (*e*_2_, *e*_4_, *e*_6_) increase from 0.5 to 0.7, the power is still lower than that in the situation when all genetic effects are at the 0.5 level. This implies that selecting relevant phenotypes is a prerequisite for maintaining the power level of a multivariate test.
7. We also investigated whether the power varies with different measurement scales, and found that the power for continuous phenotypes (*e*_1_ = *e*_2_ = 0.9, *e*_*j*_ = 0, *j* = 3, 4, 5, 6) is the highest; the power for ordinal phenotypes (*e*_5_ = *e*_6_ = 0.9, *e*_*j*_ = 0, *j* = 1, 2, 3, 4) is the next highest; and that for binary phenotypes (*e*_3_ = *e*_4_ = 0.9, *e*_*j*_ = 0, *j* = 1, 2, 5, 6) is the lowest. Such reduction in power from continuous to ordinal is relative small in comparison to the reduction from ordinal to binary. Thus, collecting phenotype data in continuous or ordinal measurement scales has the advantage of increasing statistical power.

**Table 3:** Simulation results for the empirical power with varied correlations *ρ* and genetic effect sizes (*e*_1_, …, *e*_6_). The *Latent* column is the power with the proposed method applied to 6 latent phenotypes. The *Mixed* column is the power with the proposed method applied to 6 observed phenotypes. The *Dichotomous* column is the power when the observed phenotypes are dichotomized. The *Continuous* column is the power when the phenotypes in mixed measurements are treated as continuous variables. The number of iterations is 10^6^ when all genetic effect sizes are zero and 10^4^ for other situations.

*ρ*	*e*_1_	*e*_2_	*e*_3_	*e*_4_	*e*_5_	*e*_6_	Latent	Mixed	Dichotomous	Continuous
0	0	0	0	0	0	0	0.00009	0.00008	0.00004	0.00009
0.35	0	0	0	0	0	0	0.00057	0.00024	0.00005	0.00025
0.75	0	0	0	0	0	0	0.00027	0.00007	0.00002	0.00012
0	0.5	0.5	0.5	0.5	0.5	0.5	0.96	0.91	0.77	0.91
0.35	0.5	0.5	0.5	0.5	0.5	0.5	0.84	0.73	0.49	0.73
0.75	0.5	0.5	0.5	0.5	0.5	0.5	0.51	0.36	0.19	0.39
0	0	0.7	0	0.7	0	0.7	0.94	0.88	0.68	0.88
0.35	0	0.7	0	0.7	0	0.7	0.82	0.68	0.42	0.70
0.75	0	0.7	0	0.7	0	0.7	0.36	0.20	0.08	0.25
0	0.9	0.9	0	0	0	0	0.94	0.94	0.67	0.94
0.35	0.9	0.9	0	0	0	0	0.84	0.84	0.42	0.83
0.75	0.9	0.9	0	0	0	0	0.37	0.37	0.05	0.38
0	0	0	0.9	0.9	0	0	0.95	0.71	0.68	0.74
0.35	0	0	0.9	0.9	0	0	0.85	0.43	0.41	0.49
0.75	0	0	0.9	0.9	0	0	0.37	0.05	0.05	0.09
0	0	0	0	0	0.9	0.9	0.95	0.90	0.69	0.92
0.35	0	0	0	0	0.9	0.9	0.85	0.72	0.42	0.78
0.75	0	0	0	0	0.9	0.9	0.38	0.16	0.05	0.30

### Real Data Analysis

We conducted real data analysis using the database from the Study of Addiction: Genetics and Environment (SAGE). The SAGE is a case-control study that gathered data from three large scale studies in the substance abuse field: the Collaborative Study on the Genetics of Alcoholism (COGA), the Family Study of Cocaine Dependence (FSCD), and the Collaborative Genetic Study of Nicotine Dependence (COGEND). The total number of individuals with individual level data available is 4,121. Each individual was genotyped using the Illumina Human 1M-Duo beadchip which contains over 1 million SNP markers.

The Fagerström Test for Nicotine Dependence (FTND) is a commonly adopted instrument for assessing the intensity of physical addiction to nicotine [35]. It consists of six items of which some are ordinal and the others are binary:

1. ftnd_1: How soon after you wake up do you smoke your first cigarette? (3 = within 5 minutes; 2 = 6–30 minutes; 1 = 31–60 minutes; 0 = after 60 minutes)
2. ftnd_2: Do you find it difficult to refrain from smoking in places where it is forbidden? (1 = yes; 0 = no)
3. ftnd_3: Which cigarette would you hate most to give up? (1 = the first one in the morning; 0 = all others)
4. ftnd_4: How many cigarettes per day do you smoke? (0 = 10 or less; 1 = 11–20; 2 = 21–30; 3 = 31 or more)
5. ftnd_5: Do you smoke more frequently during the first hours after waking than during the rest of the day? (1 = yes; 0 = no)
6. ftnd_6: Do you smoke when you are so ill that you are in bed most of the day? (1 = yes; 0 = no)

The items are summed to yield a total score of 0–10. The higher the total score, the more intense is the patient’s physical dependence on nicotine. Clinically, the score of 6 or higher indicates high nicotine dependency and represents individuals who would be particularly likely to benefit from tapering and/or the prescription of nicotine replacement therapy as an adjunct to standard counseling. The score of 5 or less, on the other hand, suggests low to moderate nicotine dependency and represents individuals for whom standard counseling is most appropriate. The total score, ftnd_total, or the binary variable, ftnd_total (binary), coded as 0 or 1 depending on whether ftnd_total< 6 would be popular choices for the phenotype measure. However, they have some important issues including (1) throwing away potentially important information; (2) assigning different weights to the items; and (3) choosing an arbitrary cutoff. Our proposed method, therefore, provides a new approach to conduct a multivariate test based on the original six items in mixed measurement scales.

**Fig 2:**
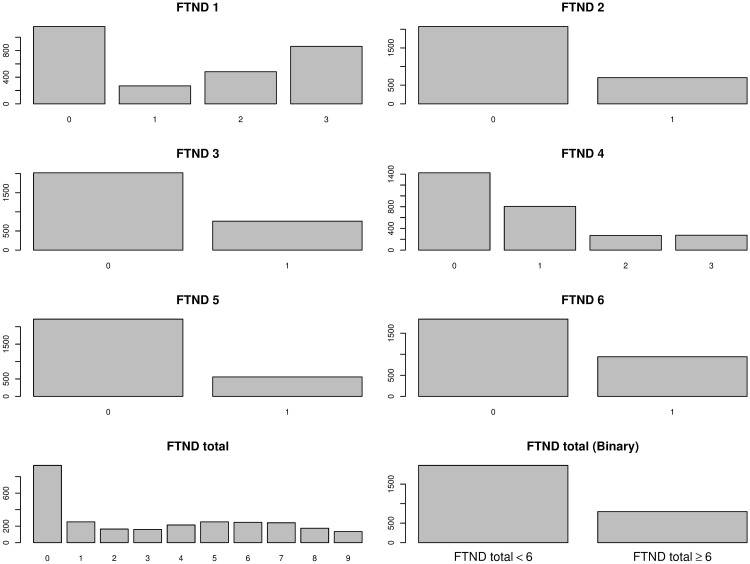
The distributions of phenotypes: FTND 1, FTND 2, …, FTND 6, FTND total, and FTND total (Binary). FTND total (Binary) is derived from FTDN total according to whether FTND total score is less than 6 or not.

From the original 4,121 individuals, we eliminated individuals whose FTND phenotype information was not available. Since a few individuals were related family members, the KING program [36] was used to identify and select unrelated individuals. The final number of unrelated individuals included in the analysis was 2,775 (1,288 males, 1,487 females). Our analysis only included 22 autosomes and the 753,238 SNP’s that passed the quality control procedures [37]. The phenotype distributions among the 2,775 individuals are presented in Fig 2 using bar-plots. Four of the FTND items (ftnd_2, ftnd_3, ftnd_5, and ftnd_6) are binary and the other two FTND items (ftnd_1 and ftnd_4) are ordinal ranging from 0 to 3. The FTND total score ranges from 0 to 9. The sample correlations among the 6 FTND items are shown in Table 4. The correlations range from 0.44 (ftnd_2 and ftnd_5) to 0.77 (ftnd_1 and ftnd_6).

**Table 4:** The correlations among the 6 FTND items.

correlation	ftnd_2	ftnd_3	ftnd_4	ftnd_5	ftnd_6
ftnd_1	0.6815	0.6758	0.7528	0.6446	0.7770
ftnd_2		0.4579	0.5895	0.4403	0.6838
ftnd_3			0.4822	0.6394	0.5294
ftnd_4				0.4452	0.6702
ftnd_5					0.5110

**Fig 3:**
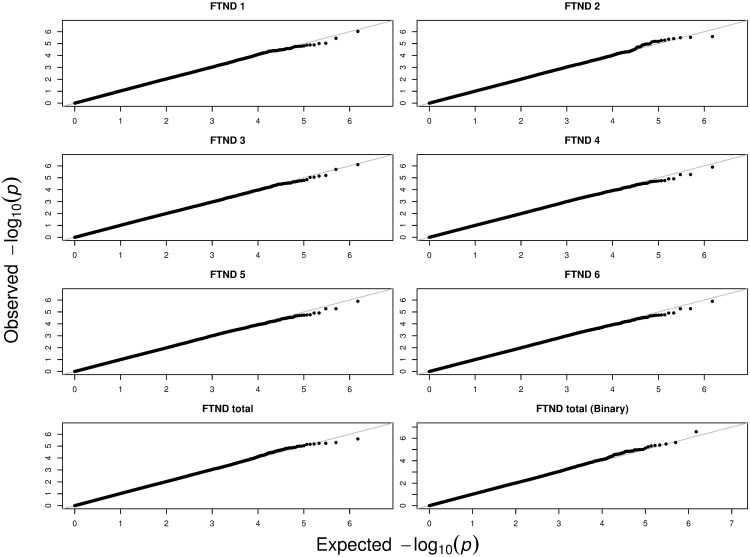
The Q-Q plots of observed *p*-values versus expected *p*-values based on the marginal tests.

**Fig 4:**
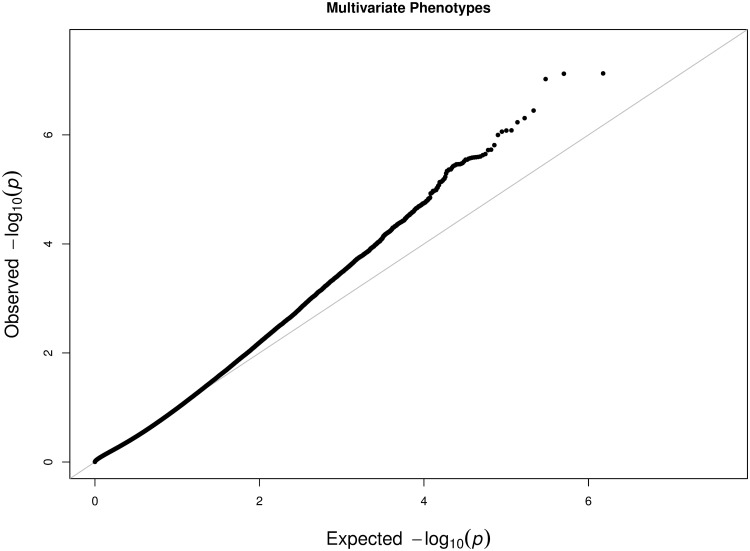
The Q-Q plot of observed *p*-values versus expected *p*-values based on the multivariate test.

We conducted marginal genome-wide association tests on the six FTND items and the two derived FTND scores. We also conducted the multivariate test based on the six FTND items. For the binary variables, the logistic regression was employed. For the ordinal variables, the cumulative logit model was used. Because the ftnd_total score was treated as a continuous variable, the linear regression was applied. For all the regression models, in addition to the genotype (coded as 0, 1, or 2), we included each individual’s age (from 18 to 74 years old), gender, and race (850 black and 1,925 white) as covariates to eliminate potential confounders. We also carried out the principal component analysis [19] to examine population stratification but did not include any principal components in the model because the first principal component perfectly matches with race (i.e. the multicolinearity issue). The marginal *p*-values are summarized using QQ-plots in Fig 3; the *p*-values based on the multivariate test using the proposed method are shown in Fig 4. The fact that more observed *p*-values are above the diagonal line in Fig 4 (in comparison to Fig 3) indicates that the multivariate test is more powerful and thus may identify more significant SNPs associated with the six FTND items.

To identify the SNPs associated with susceptibility to FTND, we set the reduced type I error rate at 10^−6^. Based on the marginal *p*-values, we identify 1 SNP (rs821722, *p* = 9.54 × 10^−7^) to be associated with ftnd_1 and 1 SNP (rs3138134, *p* = 7.94 × 10^−7^) with ftnd_3. The other four FTND items are not associated with any SNP. The derived phenotype based on ftnd_total is also not associated with any SNP. Besides, the SNP that is associated with ftnd_1 is also associated with ftnd_total (binary). On the other hand, using the proposed multivariate test, we identify 9 SNPs (rs17538699, rs17798885, rs2245261, rs4077464, rs4658846, rs4658847, rs6553017, rs7672047, rs944582) to be associated with the six FTND phenotype variables. This demonstrates that combining multiple phenotypes can increase the power of identifying markers that may not be, otherwise, chosen using marginal tests. In addition, marginal tests may identify those SNPs that only contribute to a particular phenotype. Therefore, if our goal is to identify the genes that contribute to the common risk shared by the six FTND items, the proposed method is a better approach than marginal tests.

## Discussion

In this study, we propose a new multivariate method for GWAS when the multivariate phenotypes are a mixture of continuous, binary, or ordinal variables. We use a latent response model to unify different data types for estimating correlation between phenotypes. The first phase of our method uses regression models with different link functions to accommodate different measurement scales of the phenotypes. These regression models not only enable us to evaluate the goodness-of-fit but also provide a way for adding covariates to adjust for potential confounders. The second phase of our method employs continuous latent responses to handle the correlation estimation of mixed data types. The simulation study demonstrates that our proposed correlation estimation methods have high levels of accuracy. The results also show that our approach conservatively estimates the variance of the test statistic so that the type I error rate is controlled.

We conducted a simulation study to evaluate the proposed multivariate test in terms of the type I error rate and statistical power when the observed phenotypes are in mixed measurement scales, in comparison to three competing methods: (1) the ideal analysis when the latent phenotypes are known; (2) a conventional approach that dichotomizes all phenotypes; and (3) a conventional approach treating all phenotypes as continuous. The simulation result shows that the proposed method maintains the power at the level very close to that of the ideal analysis while controlling the type I error. Furthermore, when mixed measurement scales are observed, dichotomizing all observed measurements could reduce the power by half. Although the power for treating all mixed measurement scales as continuous variables is close to that for the proposed method, this conventional approach is not recommended because fitting a linear regression model on categorical variables is both mathematically and practically questionable.

Our real data analysis using the well-known database, SAGE, in the addiction field demonstrates that conducting a multivariate test on multiple phenotypes can increase the power of identifying markers that may not be, otherwise, chosen using marginal tests. The proposed method also offers a new approach to analyzing the items rather than the total score of FTND as multivariate phenotypes in GWAS. In summary, the proposed method is a better approach than marginal tests to identify pleiotropic genes that contribute to the common liability to complex diseases such as substance use disorders.

Although the proposed method was designed to handle continuous, binary, and ordinal phenotypes, it can be extended to deal with count data. Under the framework of our two-phase approach, the first phase would employ a Poisson or negative binomial regression model to conduct a marginal test on count data; and the second phase would treat the count data as continuous in calculation of pairwise correlations, because both Poisson and negative binomial distributions can be approximated by a normal distribution based on the large sample theory [38]. The proposed method involving Kendall *τ* is also robust against deviation from a normal distribution. Nevertheless, future research is needed to further extend the method to handle zero-inflated count data such as the number of alcohol use disorder symptoms [39, 40]. It is also important to extend the proposed method to deal with nominal phenotypes such as disease subtypes. For example, Zucker (1994) [41] proposed a well-known developmental theory that classifies alcoholism into 4 subtypes: antisocial alcoholism, developmentally limited alcoholism, negative affect alcoholism, and the primary alcoholism (isolated, episodic, and developmentally cumulative).

In standard case-control studies, the proportion of cases in the sample may be much higher than that in the population. To deal with this ascertainment bias, many studies employed a liability threshold model [42] assuming an underlying latent random variable, which is normally distributed in the population and has a certain threshold that determines the disease status. Zöllner and Pritchard (2007) [43] proposed another approach to correct the ascertainment bias directly based on population prevalence of the disease phenotype and sampling scheme. They also conducted a simulation study showing that when the association test is powerful or the sample size is in thousands (applicable to our setting), the ascertainment bias is negligible and thus the correction may not be necessary. These existing methods and simulation results are, however, based on GWAS with the case-control design involving a binary phenotype. How to extend these methods to handle GWAS with multivariate phenotypes is, therefore, a very important and yet complex question for future research because the definition of “case” is unclear, especially when the phenotypes are continuous.

The method proposed in this study was designed for GWAS with independent subjects. Due to reduced costs for SNP arrays, in recent years, many family studies have collected GWAS data [44–46] so relatedness has become a new component to account for in modelling. The linear mixed model (LMM) has been used to adjust for correlation between related subjects with a univariate phenotype [47]. When multivariate phenotypes from related subjects are considered, the association test between a SNP and multivariate phenotypes needs to account for an additional level of correlation. Since the computational bottleneck is to estimate genetic correlation matrix, direct implementation of the LMM can only handle a sample size in hundreds. Thus, the computation becomes very expensive when LMM is extended to multivariate phenotypes. Zhou and Stephens (2014) [48] proposed an efficient matrix-variate linear mixed model (mvLMM) to identify pleiotropic genes while controlling for correlation among a large sample of related subjects. Theoretically, their method is applicable to any number of phenotypes. However, the complexity of their method and computational speed increase with the number of phenotypes. Specifically, th number of parameters in the mvLMM and the computational time for the EM algorithm is quadratically proportional to the number of phenotypes. Therefore, mvLMM is only applicable to a modest number of phenotypes (fewer than 10 traits). A future direction of research is to extend the proposed method to handle related subjects and compare it with mvLMM on performance.
